# Effects of a Biobased Antioxidant Gel on Meat Shelf-Life: Oxidative Stability and Color as Quality Parameters

**DOI:** 10.3390/gels11040279

**Published:** 2025-04-08

**Authors:** Olimpia Pitirollo, Edmondo Messinese, Maria Grimaldi, Davide Barbanti, Antonella Cavazza

**Affiliations:** 1 Department of Chemistry, Life Sciences and Environmental Sustainability, University of Parma, 43124 Parma, Italy; olimpia.pitirollo@unipr.it (O.P.); edmondo.messinese@unipr.it (E.M.); 2 Department of Engineering for Industrial Systems and Technologies, University of Parma, 43124 Parma, Italy; maria.grimaldi@unipr.it; 3 Department of Food and Drug, University of Parma, 43124 Parma, Italy; davide.barbanti@unipr.it

**Keywords:** gels, active spray, antioxidants, color analysis, meat, oxidative stability, shelf-life

## Abstract

Oxidative stress is one of the main factors affecting food stability; therefore, antioxidant additives are generally used as food supplements for shelf-life improvement. In this work, the use of an antioxidant gel based on natural polysaccharides was tested on different types of meat, such as hamburger, beef steak, and horse fillet. The oxidative stability was measured on minced meat by Oxitest reactor, an automated tool performing accelerated shelf-life analysis by monitoring the fat oxidation process. The primary and secondary shelf-life of the gel was evaluated by DPPH assay. The effect of the gel on meat shelf-life was examined by colorimetry providing information about the color variation (∆E) during time. Treated meats showed lower color variation compared to untreated samples. Moreover, some color coordinates were selected as markers to follow the oxidation phenomenon. In conclusion, the antioxidant gel was found to preserve meat from oxidation, increasing stability during shelf-life.

## 1. Introduction

The color of foods of both plant and animal origin derives from pigments that can be distinguished according to their polarity in lipophilic (chlorophyll: green; carotenoids: yellow, orange, red) or hydrophilic (flavonoids: yellow; anthocyanins: red, blue; betalaine: red, violet) [1]. In vegetables and fruits, pigments and dyes change qualitatively and quantitatively during the different stages of maturation [2]. Therefore, the colorimetric characteristics of foods are connected to information about their state of maturation, conservation, and possible alteration. Hence, color is an important indicator of the shelf-life of the products and of their eventual biological and technological alteration (inappropriate use of additives/dyes, incorrect heat treatments) [3,4]. Consequently, color is an important indicator of the quality of the food product, both from the point of view of the producer, who often uses it as a process parameter, and for the consumer who chooses food mainly in relation to aesthetic and chromatic characteristics [5]. These aspects are the first qualitative input that the consumer perceives and evaluates to realize the freshness and state of ripeness, creating organoleptic and sensory expectations that the consumers will find at the time of consumption.

The main factors affecting meat stability during processing and subsequent storage are lipid oxidation and deterioration due to microorganism growth [6]. The oxidative degradation of lipids and proteins causes significant loss of nutritional composition and results in protein fragmentation, amino acid crosslinking, peptide side-chain modification, and consequently, offensive odors and changing colors. Different methods to prolong meat shelf-life have been proposed by researchers, including active packaging [7,8]. The principle of active packaging consists of including active agents in the packaging which interact with meat and/or its environment by releasing antioxidant compounds in order to delay degradation due to lipid, protein, and pigment oxidation, thus extending the period that products are sensory acceptable. In particular, the use of essential oils as additive active coating for meat treatment has been proposed for their antioxidant and antimicrobial activity [7]. Several studies have reported the use of edible active packaging as an emerging technology to extend shelf-life of meat products [9,10,11]. Edible films represent an active packaging formulation typically prepared by using natural polysaccharides and enriched with antioxidant and/or antimicrobial substances, such as essential oils, or extracts from agro-industrial by-products. Indeed, Grimaldi et al. [9] already reported the preparation of active edible films based on sodium alginate enriched with different extracts obtained from agro-industrial by-products (such as artichoke, red onions, thistles) able to extend the shelf-life of meat [9]. These innovative materials prolong the shelf-life by releasing active compounds, and can be even cooked and eaten together with the food product [9].

In this context, it is important to check the quality of meat and its state of decay. The use of color as an indicator of food quality is a quick and easy parameter to measure, well related to other physico-chemical properties of the product [2,12,13]. Historically, the investigation of food colors is part of sensory analysis, which over the years has been replaced by instrumental measurements capable of performing an evaluation of food in real time and objectively [5,14]. In the RGB model, the chromatic scale is obtained from the sum, to varying degrees, of the additive primary colors: red, green and blue https://it.wikipedia.org/wiki/Blu, accessed on 2 April 2025 [12]. The sum of the three colors in equal parts constitutes white while their total absence is black; other notable colors of this type of model are yellow (red + green), magenta (red + blue) and cyan (green + blue) [12,15]. Due to its characteristics, it is a particularly suitable model in the representation and display of images in electronic devices [16]. Indeed, most devices normally use combinations of red, green, and blue to display the pixels of an image. However, this also means that it is particularly dependent on the device itself: the same image may be displayed differently when seen on two different devices, as the materials used to make the screens vary by manufacturer [16]. In addition, differences can be noted over time even in the same device, due to the natural deterioration of the product. An image can be decomposed through filters or other techniques in the basic colors that allow evaluation of the visible spectral region [17]. One of the most used spaces today is the CIELAB 1976 space [14], derived from the XYZ space, characterized by the coordinates L*, a* and b*. The parameter L* represents the brightness (i.e., the position on the vertical axis from black L* = 0 to white L* = 100), while the values of a* and b* define the chromaticity of the color [18]. The great advantage of the CIELAB system consists in the possible correlation of the distances between two points of space with the chromatic differences perceptible by the human eye. In this case, the distance (∆E) between two points is defined as the geometric distance between two points in space. For ∆E = 1, the difference between two colors is difficult to perceive by the human eyes; for ∆E = 2, the difference between two colors is clearly perceptible; for ∆E > 5 the difference is evident even without direct comparison.

As already demonstrated, the storage of food products in active packaging represents a sustainable alternative to the use of plastics, ensuring the improvement of products’ shelf-life by enhancing their oxidative stability [9,10,11]. In literature, several studies have shown that the color monitoring of food over time by measuring coordinates variation (∆E) in the CIELab color space can be considered a crucial parameter related to food quality. For instance, the typical red coloration of meat is mainly subjected to changes during storage. Indeed, oxidation phenomena lead to a significant color transition from bright red (oxymyoglobin) to brown (metmyoglobin), a process that results in a degradation of the food and reduction of the food’s appeal to consumers [19]. This transition is associated with the oxidation of myoglobin, where the formation of metmyoglobin is a key indicator of quality loss [4]. In this context, color variation (∆E) in the CIELab color space has been identified as a key parameter to evaluate color changes of food products. According to Hernández Salueña et al. [4], ∆E measurement provides a robust measure of perceptible color differences promoted by oxidation, capturing the cumulative effect of variations of the L*, a*, and b* coordinates. It has been demonstrated that a significant increase in ∆E correlates with an increased proportion of metmyoglobin, reflecting the deterioration of meat quality and the consequent reduction in consumer acceptances [4]. Moreover, during meat oxidation, the color variation generally provided a progressive increase as oxidation advanced, particularly when met-myoglobin content exceeded 20%, which is generally associated with consumer rejection [4,20]. This variation is crucial for accurately assessing meat freshness and determining the point at which the product becomes visually unappealing. Furthermore, ∆E has shown to be more sensitive to changes in redox states than individual color coordinates, emphasizing its significance as a comprehensive indicator of meat quality [20].

In this work, color analysis was used for the first time to monitor the shelf-life of meat treated with an active gel enriched with basil extract. In detail, an antioxidant gel formulation based on polysaccharides and antioxidant components contained in basil extract is proposed as an active solution capable of prolonging meat shelf-life during storage. The oxidative stability of the spray was first evaluated using Oxitest reactor measurements. Different colorimetry methods were also tested to record color coordinates and monitor color variations in both treated and untreated meat samples. This approach not only enhances monitoring accuracy but also offers an effective system to determine the performance of innovative packaging solutions in preserving meat freshness and visual appeal.

## 2. Results and Discussion

### 2.1. Oxidative Stability of the Active Aroma by Oxitest Measure

The antioxidant activity of hydro-soluble basil aroma was tested by means of an Oxitest reactor. This analysis is carried out using two pressurized chambers and allows measurement of the consumption of oxygen which reacts with fat over time. The result is the Induction Period (IP), corresponding to the steady decrease of oxygen pressure and the sample oxidation process. The longer the IP, the higher the stability against oxidation over time. This technique was selected because it allows performing measurements on the whole product without extraction nor pretreatments; therefore, it takes into account the entire effect of all the occurring compounds, including pro- and anti-oxidant molecules.

The investigation focused on a minced meat sample with a fat content of 25%, which was treated with a hydro-soluble aroma and compared with the untreated control (TQ). The IP value recorded for the TQ sample was approximately 430 ± 6 min, whereas the IP value of the treated meat was 455 ± 4. Therefore, at T0 the meat enriched with the hydro-soluble aroma provided longer IP, leading to higher oxidative stability compared to the TQ sample. Furthermore, minced meat treated with hydro-soluble aroma and stored at 4 °C after 24 and 96 h exhibited significantly longer IP values (*p* < 0.05) of 485 ± 22 and 522 ± 32, respectively.

The induction period of treated meat showed a slight enhancement of the oxidative stability over time, ranging from 5% to 17% compared to the control. In contrast, the analysis performed on control samples demonstrated a progressive reduction of IP values, reaching a value of approximately 90 min after 96 h of storage. This trend highlighted the protective effect of the hydro-soluble aroma against oxidative degradation. Notably, after storage at 4 °C for both 24 and 96 h, a significant and progressive enhancement in oxidative stability was observed, further confirming the efficacy of the treatment in mitigating lipid oxidation.

### 2.2. Gel Preparation and Antioxidant Activity

The gel was prepared as reported elsewhere [9], and then packaged in plastic spray dispensers and stored at room temperature in the dark. The shelf-life of the gel was evaluated by monitoring the antioxidant activity by colorimetric assay (DPPH assay) over six months, with measurements conducted at two-month intervals. The DPPH assay was chosen as it is recognized to be an universal method for the analysis of the total antioxidant activity in order to perform the primary and secondary shelf-life of the active gel.

Table 1 reports data collected on three separate bottles, each containing the same solution, prepared on the same day but opened at different time intervals, in order to investigate the impact of exposure on stability.

Data showed no significative differences between reduction observed during primary and secondary shelf-life of the gel in terms of antioxidant activity. After 6 months, the spray showed a reduction of approximately 70% of its antioxidant activity, and after 1 year no further significative difference was observed. This effect might be due to the mechanism of oxidation of the hydrosoluble basil aroma incorporated into the gel formulation that produces oxidized compounds and, as consequence, a reduction of antioxidant activity. Basil extract contains active compounds, such as phenolic acids, flavonoids, saponins, alkaloids, and quinones. These compounds are known to effectively scavenge free radical species, reactive oxygen species (ROS), and reactive nitrogen species (RNS), contributing to antioxidant activity that may help reduce oxidative stress [21,22]. Indeed, it is reported that antioxidants are a class of compounds able to effectively reduce a pro-oxidant, while simultaneously forming products with no or minimal antioxidant activity [23].

In this study, the spray was used during the first 4 months of its preparation in order to perform experiments on different meats and evaluate the effects of the gel on meat shelf-life.

### 2.3. Color Analysis: Correlation Between Meat Oxidation and Color Variation

#### 2.3.1. Method A Analysis

**Figure 1:**
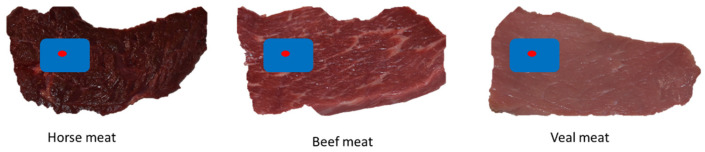
Areas submitted to image analysis with ImageJ 2 software. In blue, the “big area”, and in red, the “small area”.

The meat samples examined were horse, beef, and veal meat. Horse meat, being particularly rich in iron, is affected by a rapid change of color. As reported by Tikhonov et al. [24], the darkest color is due to the higher content of myoglobin that, when exposed to air, forms the pigment oxymyoglobin. Data were reported for untreated (TQ) and treated (TR) meat with active gel, and color was detected at times T0, T24, T48, T72, and T96. An area of the image was selected and analyzed through ImageJ 2 to characterize the color (Figure 1).

The selected area represents the part of the image that is large enough to provide most of the colorimetric information able to describe the sample. This area, highlighted in blue in Figure 1, is named the “large area”. Within this region, a significantly smaller zone, termed the “small area” and highlighted in red in Figure 1, was also analyzed to provide more localized and precise information.

**Figure 2:**
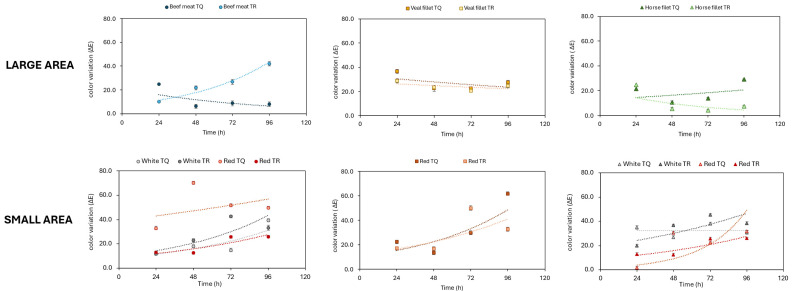
The ∆E behavior over time for horse meat, beef meat and veal meat in “big area” and “small area”.

Data collected for all types of meat showed a substantial variability in single RGB coordinates within the “large area”, with differences in the order of magnitude of approximately 100. In contrast, the “small area” demonstrated much closer tolerance among RGB coordinates, with measured minimum and maximum values differing by about 10 points. The average values from both RGB coordinates were used to calculate the color variations (∆E) over time. Figure 2, instead, displays the ∆E trend from 24 h to 96 h for all meats, highlighting how color changes progressed during the storage period.

Figure 2 shows that the data obtained from the “large areas” of all samples showed slight differences between TR and TQ samples. On the other hand, the differences observed in the “small areas” are much more pronounced. Noticeably, concerning the beef sample, in the area highlighted in red, the untreated meat has a ∆E of around 70 after 48 h, while the meat wrapped in film has a much lower ∆E of around 20. Overall, spray treatment effectively reduces color variation of the meat, thereby helping to extend meat shelf-life. This would suggest that the bio-based antioxidant gel formulation successfully preserves the visual quality of the product during storage. However, Method A for colorimetric data analysis demonstrated several limitations that affected the reliability of the provided information. These critical issues arise primarily from both the heterogeneous nature of the analyzed subject and the acquisition methodology employed, leading to variability and inconsistency in the colorimetric data.

Food matrices are inherently heterogeneous, presenting challenges for accurate color analysis. In the test conducted, both red and white portions contributed to define the overall color of the sample, impacting on consistent and representative measurements. To accurately assess the color of heterogeneous matrices, measurements should be localized and precise to effectively predict specific deterioration processes. In “small areas”, the variability of RGB coordinates decreases considerably, resulting in more stable and reliable data. Therefore, adopting standardized data acquisition methodologies is essential to minimize external variability. A practical approach may involve fixing the camera and sample position, consistently capturing images from the same location in the same conditions and evaluating color changes within the same sampled area. This methodology reduces measurement inconsistencies and enhances the accuracy and repeatability of color evaluations.

In conclusion, data obtained by Method A were unstable and only provided a rough indication of the phenomenon under investigation. Due to its lack of reliability and precision, it cannot be considered suitable for accurate or punctual analysis, unless the samples’ surfaces are characterized by very good homogeneity.

#### 2.3.2. Method B Analysis

**Figure 3:**
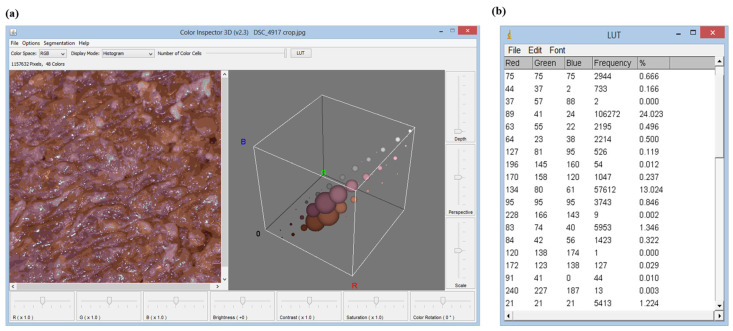
Color analysis with Method B. (**a**) Selected area of analysis; (**b**) output: RGB coordinates and % frequency.

Method B was used to measure the color variation of untreated (TQ) and treated with gel solution (TR) beef hamburgers at times T0, T24 and T48. This method exploits the “Color inspector 3D” tool to obtain information concerning the frequency of each color coordinate extracted from a target area of the picture, as displayed in Figure 3.

**Figure 4:**
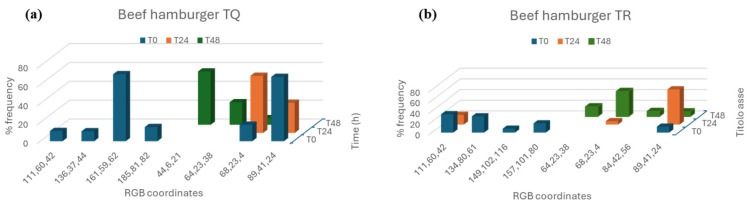
Frequencies (expressed as percentages) of RGB coordinates related to the hamburger over time: (**a**) TQ sample; (**b**) TR sample.

Therefore, Method B can relate RGB coordinates obtained with a frequency (expressed as percentage) (see Figure 3b), indicating the prevalence of each color in the image (Figure 3a). Figure 4, in contrast, presents data obtained using Method B for both untreated (Figure 4a) and treated (Figure 4b) hamburger. This type of approach has the advantage that it is not disturbed by surface inhomogeneity, as occurs with Method A.

The analysis of RGB coordinates verified that the samples at T0 have some coordinates which are no longer visible after 24 h (T24), while others remain persistent over time but with different % frequencies (Figure 4). Finally, after 48 h, there are some RGB coordinates not observed at T0 and T24 (Figure 4). This means that deterioration phenomena responsible for the color change of the matrices has occurred. Method B can be an excellent approach for monitoring and analyzing complex matrices using low-cost and easily available tools. However, a punctual calibration of the method is required, and as an interesting prospect, it would be useful to identify marker colors of a specific food matrix (system) which can give information about storage time.

#### 2.3.3. CIELab Space

**Figure 5:**
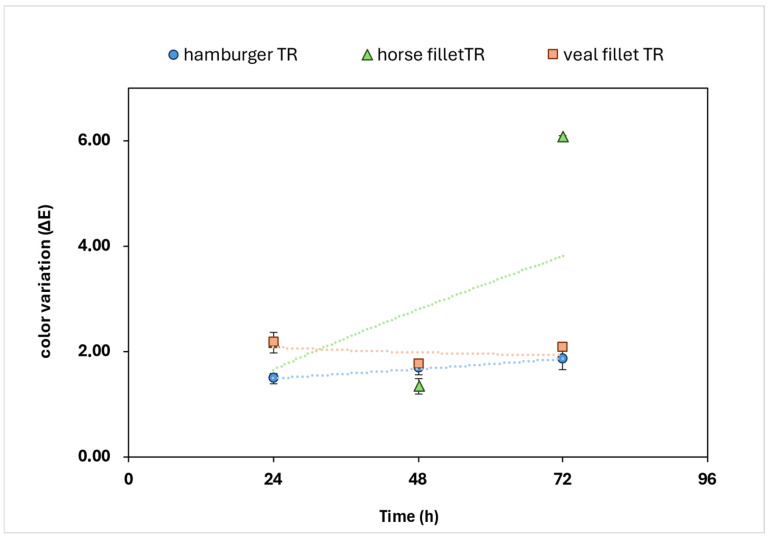
Graphical representation of color coordinate variation (∆E) of treated meat samples during storage.

The evaluation of color changes in meat samples (horse meat, veal meat and beef hamburgers) was carried out by color variation (∆E) measurement in CIE L*a*b* color space. The evaluation of color coordinate differences associated to this parameter is generally well related with the human ability to distinguish colors [4]. In Table 2, the color variation of treated (TR) and untreated (TQ) meat are reported. For horse meat, a stronger increase was observed for ∆E at T72 of TR and TQ compared to initial stages, that present stable values (Figure 5).

Although the values of the TR meat samples remain lower only at T24, statistical analyses did not confirm significant differences in treatment, and this is probably due to the accelerated formation of meta-myoglobin, compounds related to browning phenomena, typical in horse meat.

For the veal fillet, over the entire period of monitoring color changes, Figure 5 clearly displays that the TR trend was steadily lower than the TQ sample. The statistical analysis shows significant differences between the values of the two treatments, suggesting that the bio-based antioxidant treatment on veal fillet reduced the color changes induced by oxidation phenomena.

In the case of the beef hamburger samples, the antioxidant treatment induced significant effects in terms of reduced color variation of the sample during storage time (*p* < 0.05) (Table 2).

As discussed above, horse meat at the end of color monitoring (T72) presented a value that was three times higher in terms of color variation (∆E), suggesting that the application was not effective on this type of matrix. The higher iron and myoglobine protein contents are probably responsible for the fastest browning and decay of color coordinates. In future work, it might be interesting to increase the antioxidant content for the application of such critical matrix samples or to evaluate a possible synergic use of compounds that can reduce the effects of oxidative stress on meat. Values referring to veal meat and hamburger present differences significantly lower compared to horse meat. Similar values of ∆E recorded at all the three stages do not show significant differences, suggesting that the treatment carried out is suitable for the extension of shelf-life during storage.

## 3. Conclusions

This research directs attention to the possible use of a very rapid analytical method based on a colorimetric assay to monitor meat storage and to its application to evaluate the effects of natural antioxidants proposed to preserve products from deterioration, thus extending shelf-life. The proposed techniques, such as colorimetry and Oxitest, allow estimation of the antioxidant effect on a system represented by a whole product instead of one usually measured by chemical analyses after complex extraction procedures. The main advantage of these analytical techniques is that the samples are analyzed without pretreatments.

The goal of the present work as only a preliminary approach was to compare two different acquisition methods for image analysis and to estimate the efficiency of both procedures to check the effect of the preservative formulation. The results of Method A showed that the color of the control samples was definitely affected over a few days, whereas treated samples kept a more stable color, except for horse fillet. The use of the tool “Color inspector 3D” of the software Image J 2 (Method B) suggests a potential deeper investigation since the measure of frequency through a 3D color scan allows the presence of some colors to be proposed as markers, thus opening new possibilities of development for future prospects. For this reason, Method B is recommended for the analysis of acquired images.

Of course, for a complete evaluation it is mandatory to complete the study by considering microbiological tests in order to check the state of the meat from a hygienic point of view and to explore a possible correlation between color indexes and microbial contamination.

## 4. Materials and Methods

### 4.1. Chemicals and Samples

Water (MilliQ), sodium carbonate, sodium alginate, calcium carbonate, glycerol, and d-(+)-gluconic acid-σ-lactone (GDL) were purchased from Sigma Aldrich (Steinheim, Germany).

The aroma and meat samples (beef hamburger, veal fillet, horse fillet, beef fillet) were commercially available and provided, and they were stored at a refrigerating temperature of 4 °C by Greci Industria Alimentare spa (Ravadese, Parma, Itay).

### 4.2. Gel Preparation

Gel formulations were prepared following a previously set up protocol [9]. Briefly, a 1% *w*/*v* solution of sodium alginate was prepared in 1 L distilled water at 70 °C under vigorous stirring. Glycerol was added at an amount of 0.3 g/g of sodium alginate. In a different beaker, a suspension of CaCO_3_ (0.04 g/g of alginate) and GDL (5.4 g/g of CaCO_3_) was prepared in 100 mL of distilled water (pH around 5.0). The suspension was then added to the alginate solution under vigorous stirring and kept at 40 °C for 30 min. A hydro-soluble aroma based on basil extract was provided by the company Greci Industria Alimentare spa (Ravadese, Parma, Itay), was added (2.5% *w*/*v*) to the mixture, and gently stirred at room temperature for 15 min. The formulation was packaged in suitable bottles to produce a spray solution.

### 4.3. DPPH Assay on Active Gel

The antioxidant activity of the gel was monitored for 6 months, assessing the DPPH assay every 2 months (T0 = day of preparation, T1 = 2nd month, T2 = 4th month, T3 = 6th month, T4 = 1 year).

The DPPH assay was based on the molecular scavenger activity of the radical DPPH•. Each class of molecules occurs in different mechanisms of oxidation, such as scavenging reactive oxygen species, converting hydroperoxides to non-radical species, absorbing UV radiation, deactivating singlet oxygen or, in some cases, binding of metal ions.

The protocol followed for DPPH assay was reported by Pasqualone et al. [25] with some modifications. An amount of 1.5 mL of freshly prepared DPPH solution in 60 μM methanol was added to 500 μL of the active spray. After 30 min under vigorous stirring, the mixture was filtered through a 0.45 µm PTFE filter, and the absorbance of the solution was measured at 515 nm through UV–VIS spectrophotometry (Thermo Scientific^TM^ Evolution^TM^ 201/220, Milan, Italy). Methanol was used as a blank. The absorbance of the DPPH solution was recorded at the same wavelength but at t = 0 min. The antioxidant activity was expressed as percentage of scavenging capacity of the DPPH radical and calculated as follows:

TAC (%) = (A_r_ − A_s_)/A_r_

where A_r_ is the absorbance of DPPH solution at t = 0 min, and A_s_ is the absorbance of the sample.

### 4.4. Oxitest

The antioxidant activity of hydro-soluble aroma and of minced meat was tested by means of the Oxitest reactor (Velp Scientifica, Usmate MB, Italy).

This device is composed of two reactors and permits acceleration of the samples oxidation process through temperature and oxygen pressure increase. The analysis measures the consumption of oxygen which reacts with fat over time. The longer the IP, the higher the stability against oxidation over time. In detail, 30 g of meat dough, was placed directly inside the three titanium sample holders, in each reaction chamber, using a spatula. The same procedure was followed for the sample containing the hydro-soluble aroma (5% *w*/*w*). Oxygen pressure was set at 6 bars, and temperature at 90 °C, according to the analytical conditions set by Velp Scientifica during the validation studies performed on different food products (IQ/OQ Oxitest Manual, Velp Scientifica). The system measures the absolute pressure change inside the chambers and monitors the oxygen uptake by the reactive components occurring in the sample. An Induction Period (IP), expressed in minutes, corresponds to the time at which a sudden change in the rate of oxidation is detected. At the end of the oxidation tests, the IP of every run is calculated by the software OXISoft^TM^. The analysis was also performed on the minced meat dough stored at 4 °C, after addition of the hydro-soluble aroma at 24 and 96 h.

### 4.5. Sample Treatment

Each slice (about 15 × 6.5 × 1.5 cm^3^, l × l × h) and each hamburger (15 × 1.5 cm^2^, l × h) was treated with 10 sprays per side, corresponding to about 0.02 mL /cm^2^. The samples were dispensed at a 5 cm distance between bottle spray and samples at room temperature. Shelf-life of meat, stored at a temperature of 4 °C covered with an aluminum foil, was checked from T0 to T96h, performing analyses every 24 h. One control sample for each meat type was stored under the same conditions.

### 4.6. Image Acquisition

Images were acquired using a Nikon D5600 camera (Nikon, Torino, Italy) with the following settings: sensibility ISO-100; focal distance of 48 mm; color filter type RGB; Flash ON, resolution 300 dpi. The samples were photographed immediately after preparation and from T0 to T96 every 24 h.

### 4.7. Image Analysis

**Figure 6:**
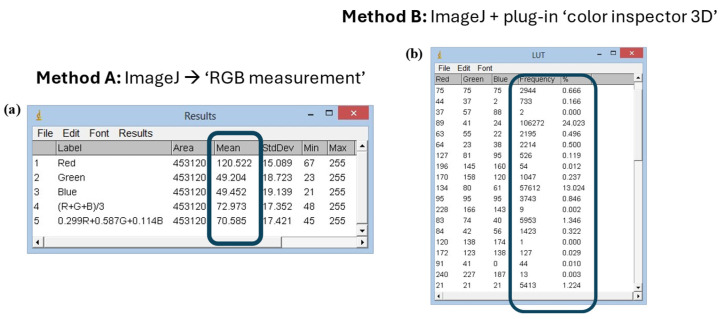
The information from ImageJ 2 software using two methodologies: (**a**) Method A and (**b**) Method B.

Two different methods were used to analyze pictures of meat samples. Both of them use the ImageJ software, but Method B involves the use of the additional tool “Color inspector 3D” that performs analysis on restricted areas of the pictures, whereas Method A allows the image analysis of the widest area of the pictures. Method A was used to analyze the color of the treated and untreated samples from T0 to T96 every 24 h. Method B was used to analyze the color of the treated and untreated beef hamburgers from T0 to T48h every 24 h (Figure 6).

In Method A, the color analysis was carried out using the “RGB Measure” command of the ImageJ software which allows obtaining the colorimetric information in the RGB field of the image under analysis (Figure 6a). The pictures acquired by the camera could be analyzed completely or in a portion of them. The color variations occurring in the considered time intervals were evaluated using the Euclidean distance between each three coordinates (red, green and blue). The evaluation of the colorimetric coordinates was then carried out by analyzing the data in Microsoft Excel.

Method B involved the re-processing of images through “Color inspector 3D”, an additional tool of ImageJ software. It allows transporting the information present in the RGB image in the different colorimetric systems and extracting quantitative information about the percentage of each color in the selected area (Figure 6b). Method B, therefore, obtains information about the most frequent color coordinates (frequency %) present in the analyzed system.

### 4.8. Colorimetric Analysis

Color was evaluated on treated and untreated horse meat, beef meat and beef hamburgers. Color was measured using a colorimeter that works in CIE L*a*b* color space (TECHKON Spectrodens, Königstein, Germany). The instrument is equipped with a LED light source. The measurement aperture was 3 mm, and two optical configurations were adopted (0° and 45°). The spectral range used was 400–700 nm in 10 nm steps, with a spectral resolution of 10 nm and the pixel distance sensor < 3 nm. Before the measurement, the colorimeter was calibrated using a white reflectance standard supplied by the manufacturer. A customized perforated jig was used to record the color coordinates in the same points allowing repeatable positioning. Three measurements of color coordinates were recorded, and the software adopted was TECHKON Spectro Connect 2.5.

Color variation (∆E) in CIE L*a*b* color space was calculated as:

$$∆E_{ab}=\sqrt{{\left(L_{2}-L_{1}\right)}^{2}+{\left(a_{2}-a_{1}\right)}^{2}+{\left(b_{2}-b_{1}\right)}^{2}}$$

where (*L*_2_ − *L*_1_), (*a*_2_ − *a*_1_), (*b*_2_ − *b*_1_) are the difference in terms of lightness, green–red and blue–yellow coordinates, respectively.

### 4.9. Statistical Analysis

For statistical analysis, normal distribution and variance homogeneity were previously tested (Shapiro–Wilk test). Analysis of variance (ANOVA) and pair-wise comparisons were performed by means of Microsoft Office Excel. Results are expressed as mean ± standard error of the mean (SEM).

## Figures and Tables

**Table 1:** %TAC reduction of the active gel over 6 months during primary and secondary shelf-life (data expressed as mean ± standard deviation).

	T0	2nd Month	4th Month	6th Month	1 Year
Bottle 1	100.0%	−2.0% ± 1.0	−5.0% ± 1.0	−70.0% ± 2.0	−75.0% ± 2.0
Bottle 2	100.0%	n.d.	−5.0% ± 1.0	−72.0% ± 2.0	
Bottle 3	100.0%	n.d.	n.d.	−71.0% ± 2.0	

n.d. (=not determined) since the bottle was not opened.

**Table 2:** Color coordinate variation (∆E) of untreated (TQ) and treated (TR). Samples during storage at 4 °C. Letters are referred to statistical significance.

	Hamburger		Veal Fillet		Horse	
Time	TQ	TR	TQ	TR	TQ	TR
24 h	2.61 ± 0.08 ^a^	1.63 ± 0.13 ^b^	2.77 ± 0.10 ^a^	1.91 ± 0.20 ^b^	3.35 ± 0.21 ^a^	1.41 ± 0.83 ^b^
48 h	2.77 ± 0.22 ^a^	1.91 ± 0.21 ^b^	2.81 ± 0.11 ^a^	1.94 ± 0.16 ^b^	2.46 ± 0.15 ^a^	1.53 ± 0.17 ^b^
72 h	3.35 ± 0.33 ^a^	1.41 ± 0.15 ^b^	2.97 ± 0.20 ^a^	1.92 ± 0.21 ^b^	3.74 ± 0.13 ^a^	6.03 ± 0.19 ^b^

## Data Availability

The raw data supporting the conclusions of this article will be made available by the authors on request.
